# National point-prevalence survey of healthcare-associated infections and antimicrobial use: UK-PAS/UKHSA joint call to action for all paediatric services

**DOI:** 10.1093/jac/dkad265

**Published:** 2023-08-23

**Authors:** Samuel Channon-Wells, Jocelyn Elmes, Berit Muller-Pebody, Orlagh McGarrity, Faye Chappell, Simon B Drysdale, Diane Ashiru-Oredope, Sanjay Patel, Alicia Demirjian

**Affiliations:** Section of Paediatric Infectious Disease, Department of Infectious Disease, Imperial College London, London, UK; UK Paediatric Antimicrobial Stewardship Network, Birmingham, UK; Healthcare-Associated Infection (HCAI), Fungal, Antimicrobial Resistance (AMR), Antimicrobial Use (AMU) and Sepsis Division, United Kingdom Health Security Agency (UKHSA), London, UK; Healthcare-Associated Infection (HCAI), Fungal, Antimicrobial Resistance (AMR), Antimicrobial Use (AMU) and Sepsis Division, United Kingdom Health Security Agency (UKHSA), London, UK; UK Paediatric Antimicrobial Stewardship Network, Birmingham, UK; Department of Pharmacy, Great Ormond Street Hospital, London, UK; Department of Paediatric Infectious Diseases and Immunology, Evelina London Children's Hospital, London, UK; UK Paediatric Antimicrobial Stewardship Network, Birmingham, UK; Centre for Paediatric and Neonatal Infection, St George’s, University of London, London, UK; Healthcare-Associated Infection (HCAI), Fungal, Antimicrobial Resistance (AMR), Antimicrobial Use (AMU) and Sepsis Division, United Kingdom Health Security Agency (UKHSA), London, UK; UK Paediatric Antimicrobial Stewardship Network, Birmingham, UK; Department of Paediatric Infectious Diseases and Immunology, Southampton Children's Hospital, Southampton, UK; UK Paediatric Antimicrobial Stewardship Network, Birmingham, UK; Healthcare-Associated Infection (HCAI), Fungal, Antimicrobial Resistance (AMR), Antimicrobial Use (AMU) and Sepsis Division, United Kingdom Health Security Agency (UKHSA), London, UK; Department of Paediatric Infectious Diseases and Immunology, Evelina London Children's Hospital, London, UK; Faculty of Life Sciences and Medicine, King’s College London, London, UK

## Abstract

The negative impact of high antimicrobial use (AMU), antimicrobial resistance and healthcare-associated infections (HCAIs) on children is concerning. However, a lack of available paediatric data makes it challenging to design and implement interventions that would improve health outcomes in this population, and impedes efforts to secure additional resources. The upcoming 2023 national point-prevalence survey of HCAIs and AMU in hospitals, led by the UK Health Security Agency, is an opportunity to collect valuable information, which will enable healthcare providers and policy makers to optimize antimicrobial stewardship and infection prevention practices in all populations, including children. These data will facilitate benchmarking and sharing of best practice, internally, nationally and internationally. This is a joint call to action asking all healthcare professionals—particularly in paediatrics—to nominate a lead for their institution and participate in this survey, to ensure appropriate paediatric representation, and help protect children from these growing threats.

## Introduction

September 2023 will see the launch of the first national point-prevalence survey (PPS) of healthcare-associated infections (HCAIs) and antimicrobial use (AMU) in UK hospitals since 2016. This work will provide vital data to guide both national and local antimicrobial stewardship (AMS) and infection prevention and control strategies. Active participation from the paediatric community is essential to ensure children are represented in this important initiative in the fight against antimicrobial resistance (AMR). Although we recognize that collecting PPS data is labour intensive, we urge all our healthcare colleagues looking after children to champion this initiative in your workplace.

## National and international benchmarking

Previous PPSs coordinated across Europe by the ECDC and nationally by PHE [now the UK Health Security Agency (UKHSA)] have enabled researchers and policy makers to compare key HCAI and AMU indicators across Europe, including the UK.^1^ The most recent survey, performed in 2016/17, demonstrated wide variation by country, highlighting the need for data collection and benchmarking at the national level. A reiteration of the survey, led by the UKHSA, will launch in September 2023 in England and Wales. This will provide a comprehensive overview of performance and practices nationally, using a validated methodology that has been shown to enhance site-specific and regional AMS strategies that could improve patient care.^2^ The survey is being conducted shortly after the recent ECDC survey (April 2023), with a protocol allowing for relevant comparative analysis internationally. Unless nurses, doctors, pharmacists, microbiologists and other professionals caring for children contribute their time and data to this project, there is a real risk that children will be forgotten.

## The need for paediatric-specific data

The use of antimicrobial agents in early life has been associated with diverse long-term conditions, including AMR and disruption of the microbiota.^3–5^ The consequences of drug-resistant infection in children and young people have potential for higher cumulative long-term impact on QALYs and disability-adjusted life years than in adults. Differences in infectious disease transmission and susceptibility make many children vulnerable to HCAIs, especially neonates, children on ICUs and those with malignancy. Collecting and studying data on HCAIs and AMU in children allows us to proactively design and implement Infection Prevention and Control (IPC) interventions targeted to children and newborns.

Similarly, these data will identify prescribing patterns specific to this population, enabling us to tailor prescribing practices and AMS programmes, with the aim of minimizing unnecessary antimicrobial exposures and reducing the risk of AMR. Previous data from the ECDC surveys and other sources show that hospitalized children are high recipients of antimicrobials, especially those aged under 5 years or in paediatric or neonatal ICUs.^1,6^ Despite this, there remains a lack of robust, reliable AMR and AMS data in children nationally and globally.^7^ Maximizing participation from paediatric services in the upcoming UKHSA survey is the best way to fill important gaps in our current data and knowledge.

## Benefits for children and young people

One of the key limitations we acknowledge of PPS methodologies is the time-consuming data collection. However, there are many substantial benefits on a population level to conducting this survey, which we believe necessitate this cost.

The two primary uses of this data will be: providing healthcare organizations with robust, comparable data to drive quality improvement programmes and promote patient safety; and providing policy makers with an evidence base for public health strategy. If policy makers understand the specific burden of HCAIs and antimicrobial consumption in children, then resource allocation, guideline development, and national interventions can be tailored to address the specific needs of children. Paediatric-specific data are particularly needed, as current paediatric AMU estimates rely on metrics unsuitable for children, and hence are likely inaccurate.^8,9^ The UKHSA PPS will allow a deep dive into the appropriateness of antimicrobial prescribing at a granular level and provide a real starting point for better benchmarking using accurate, robust data.

Funding for paediatric AMS and IPC activity in the UK is challenging,^10^ and is particularly stretched in the current political and healthcare climate. The issue is most starkly observed in neonatal ICUs, where HCAI outbreaks are commonplace,^6,11,12^ and antimicrobial use is very high,^1^ providing a nidus for development of AMR infections and associated long-term adverse health outcomes.^13^ Participation in these large-scale collaborative programmes will provide additional evidence of the benefits of paediatric AMS activity, both clinical and financial, helping paediatric services to argue for additional paid AMS time.

Participation in this survey will contribute to fostering increased collaboration between healthcare professions and researchers across local areas and services, straddling secondary and tertiary care centres, and research institutes. This will enable rapid identification and sharing of best practices, whilst providing data that will form the foundations for future research. Participation from non-academic and smaller local hospitals (District General Hospitals in the UK), where funding for paediatric AMS services is even more restricted, is particularly encouraged.

## The rights of children

By embracing this initiative, healthcare providers can contribute significantly to addressing AMR and HCAIs in children. Children and young people have the ‘right to care that fits their needs’.^14^ We must unite as healthcare professionals, administrators and policymakers to prioritize paediatric data collection, as it holds the key to a healthier and safer future for our children.
